# Carbon–Nitrogen Metabolism Associated with Appearance Quality in Superior and Inferior Grains of Soft and Non-Soft Japonica Rice in Southern China

**DOI:** 10.3390/plants15081155

**Published:** 2026-04-09

**Authors:** Xi Chen, Jianghui Yu, Ying Zhu, Guodong Liu, Guangyan Li, Fangfu Xu, Qun Hu, Jiale Cao, Hongcheng Zhang, Haiyan Wei

**Affiliations:** 1 Jiangsu Key Laboratory of Crop Genetics and Physiology/Jiangsu Key Laboratory of Crop Cultivation and Physiology, Agricultural College, Yangzhou University, Yangzhou 225009, China; 15148856902@163.com (X.C.);; 2 Jiangsu Co-Innovation Center for Modern Production Technology of Grain Crops, Yangzhou University, Yangzhou 225009, China; 3 Research Institute of Rice Industrial Engineering Technology, Yangzhou University, Yangzhou 225009, China

**Keywords:** soft rice, superior and inferior grains, carbon and nitrogen metabolism, grain filling, appearance quality

## Abstract

To investigate the differences in carbon and nitrogen metabolism between superior and inferior grains of southern soft and non-soft japonica rice and their relationships with appearance quality, the metabolic characteristics and appearance quality of superior and inferior grains during the grain-filling stage were compared between the two rice types. The results showed that, compared with non-soft japonica rice, the activities of AGPase and GBSS in superior grains of soft rice were significantly lower, whereas the activities of SSS, SBE, and DBE were significantly higher. The amylose content decreased by 32.68–44.72%, while amylopectin increased by 7.27–10.73%. The limitation in carbon metabolism was more pronounced in inferior grains, and the non-structural carbohydrate content was 9.33–17.33% lower than that in superior grains. In terms of nitrogen metabolism, GS activity decreased whereas GOGAT activity increased in superior grains, resulting in a 6.28–8.38% increase in protein content. The protein content of inferior grains was 1.75–6.44% higher than that of superior grains. In addition, the chalky grain rate and chalkiness degree of superior grains in soft rice were 79.00–481.03% higher than those in non-soft japonica rice, while the increases in inferior grains ranged from 67.51% to 136.31%. Correlation analysis indicated that the chalky grain rate of superior grains was positively correlated with starch content during the early grain-filling stage, whereas the chalkiness degree of inferior grains was positively correlated with protein content. These results suggest that differences in carbon and nitrogen metabolism between grain positions are closely associated with the formation of appearance quality.

## 1. Introduction

Rice is one of the most important staple crops in the world, and its production is vital not only for food security but also for the quality of human life [1]. In recent years, with the improvement of living standards, the demand for high-quality japonica rice has been steadily increasing. Soft japonica rice with low amylose content, which has been widely promoted in southern rice-growing regions, is favored by the market due to its soft and delicate texture after cooking and its good palatability even when cooled. It has become an important type in the production of high-quality japonica rice [2]. However, in practical production and commercialization, soft japonica rice generally exhibits appearance quality problems such as low endosperm transparency and high chalkiness [1], which have become important factors limiting the improvement of commercial grade and market competitiveness of soft japonica rice. Therefore, while maintaining its superior eating quality, systematically elucidating the underlying mechanisms responsible for the formation of appearance quality in soft japonica rice has become an urgent scientific issue that needs to be addressed.

Rice appearance quality, particularly chalkiness and transparency, fundamentally depends on the accumulation characteristics and spatial distribution of starch and protein within the endosperm [3]. Carbon metabolism plays a crucial role in starch biosynthesis and is closely associated with the compactness and optical transparency of the endosperm [4,5,6]. Nitrogen metabolism also plays an important role in endosperm transparency and chalkiness by regulating protein synthesis [7,8,9]. It is worth noting that appearance quality varies significantly among different grain positions within the panicle; typically, inferior grains exhibit higher chalkiness and lower transparency [10,11]. These positional differences are closely associated with the timing and intensity of nutrient acquisition during grain filling [11,12]. Therefore, the spatiotemporal differences in carbon and nitrogen metabolism among different grain positions are key factors leading to variations in appearance quality [13,14,15]. In terms of carbon metabolism, non-structural carbohydrates transported from vegetative organs, such as the stem and sheath, to the grains are converted into starch through the coordinated action of key enzymes, including ADP-glucose pyrophosphorylase (AGPase), granule-bound starch synthase (GBSS), and soluble starch synthase (SSS) [16,17]. The activities of key enzymes involved in carbon metabolism, together with substrate supply, are closely associated with the rate of starch synthesis and its final accumulation [16,17]. In terms of nitrogen metabolism, nitrogen transported from vegetative organs to the grains is assimilated into amino acids under the action of key enzymes, such as glutamine synthetase (GS) and glutamate synthase (GOGAT), and subsequently participates in the synthesis of storage proteins [18,19]. The activities of key enzymes involved in nitrogen metabolism are therefore closely related to the protein synthesis capacity of the grains [18,19]. More importantly, carbon and nitrogen metabolism interact in a complex manner. When nitrogen metabolism in the grains is highly active, carbon metabolism may be constrained because of competition for substrates and energy, resulting in relatively reduced starch accumulation and alterations in the protein-to-starch ratio [20]. This dynamic balance between carbon and nitrogen metabolism collectively shapes the final accumulation patterns of starch and protein in the endosperm.

Although previous studies have investigated the effects of grain position on rice appearance quality, most have mainly focused on phenotypic variation among grain positions or analyses limited to a single cultivar [12,21]. For soft japonica rice, a special type of rice, it remains unclear whether its appearance quality is associated with its distinctive characteristics of carbon and nitrogen metabolism, and how differences in carbon and nitrogen metabolism among grain positions regulate starch and protein accumulation, thereby affecting appearance quality. A systematic understanding of these mechanisms is still lacking. Therefore, this study selected representative soft japonica rice cultivars (Nanjing 5718 and Nanjing 9108) and non-soft japonica rice cultivars (Huaidao 5 hao and Huajing 5 hao) from the southern rice-growing region of China as experimental materials. The differences in carbon and nitrogen metabolism between superior and inferior grains during the grain-filling period were systematically analyzed. In addition, the dynamic accumulation of starch and protein and their relationships with appearance quality were examined to explore their intrinsic associations. Through a direct comparison between soft and non-soft japonica rice cultivars, this study aims to reveal the regulatory mechanisms by which differences in carbon and nitrogen metabolism during grain filling influence the formation of appearance quality within the panicle, thereby providing a theoretical basis for improving the appearance quality of soft japonica rice and for optimizing its corresponding cultivation practices.

## 2. Materials and Methods

### 2.1. Experimental Site and Test Materials

This research was conducted at the experimental farm affiliated with Yangzhou University, located in Yangzhou City, Jiangsu Province, China (geographical coordinates: 119°42′ E longitude, 32°39′ N latitude), during the year 2023. The soil in the experimental field was classified as sandy loam, with the following nutrient contents: 0.13% total nitrogen, 165.9 mg kg^−1^ of alkali-hydrolyzable nitrogen, 33.7 mg kg^−1^ of Olsen phosphorus, 77.6 mg kg^−1^ of exchangeable potassium, and 30.9 g kg^−1^ of organic matter.

The rice cultivars selected for this study included two premium soft rice varieties, NJ5718 (Nanjing 5718) and NJ9108 (Nanjing 9108), as well as two non-soft rice cultivars, HD5 (Huaidao 5 hao) and HJ5 (Huajing 5 hao). These cultivars differed in growth duration, spikelet number per panicle, and 1000-grain weight, thereby providing contrasting agronomic backgrounds for evaluating positional variation in chalky appearance. Detailed information is provided in Table 1.

### 2.2. Experimental Design

The field experiment was arranged in a randomized complete block design with three replicates (blocks). Each plot covered an area of 36 m^2^ (6 m × 6 m). Rice seeds were initially sown in a nursery on 19 May and later transplanted on 11 June, with four seedlings planted per hill, at a row spacing of 30 cm and a plant spacing of 12 cm. A total of 270 kg ha^−1^ of nitrogen was applied, distributed across the base, tillering, and panicle stages in a 35:35:30 ratio. The tillering fertilizer was applied seven days after transplantation, and the panicle fertilizer was applied at the fourth leaf stage. The nutrient mix followed a nitrogen-to-phosphorus-to-potassium ratio of 2:1:2. Phosphorus was applied exclusively as a base fertilizer, while potassium was evenly distributed before tillering and at the jointing stage. Irrigation practices, as well as weed and pest control measures, followed local agronomic recommendations throughout the rice growth period.

### 2.3. Measurement Items and Methods

#### 2.3.1. Leaf Photosynthetic Rate and SPAD

Measurements were conducted at 10, 20, 30, and 40 days after flowering, as well as at maturity, using a LI-6400XT portable photosynthesis system [22]. All measurements were performed between 9:00 and 11:00 a.m. on clear days, with data recorded after the readings stabilized. The SPAD values of the upper three leaves were measured using a SPAD-502 chlorophyll meter (Konica Minolta, Tokyo, Japan). Multiple positions on each leaf were measured, and the average value was calculated.

#### 2.3.2. Non-Structural Carbohydrates (NSC) and Nitrogen

Rice plants were sampled at the heading stage, 10, 20, 30, and 40 days after flowering, and at maturity. The plants were separated into stem-sheath, leaves, and panicles. The grains within a panicle were classified into superior and inferior grains according to their position on the panicle and branch type. Superior grains were defined as those located on the primary branches in the upper one-third of the panicle, whereas inferior grains were defined as those located on the secondary branches in the lower one-third of the panicle [13]. Samples were deactivated at 105 °C for 30 min, then oven-dried at 80 °C to a constant weight before being weighed. The dried samples were ground and passed through a sieve for further analysis.

Non-structural carbohydrate (NSC) content was determined according to the instructions of the plant soluble sugar/starch assay kit (Suzhou Keming Biotechnology Co., Ltd., Suzhou, China). Nitrogen content was measured using an automatic Kjeldahl nitrogen analyzer (Kjeltec 8200, Foss, Hillerød, Denmark).

#### 2.3.3. Carbon and Nitrogen Metabolism-Related Enzyme Activities

Single stems that flowered on the same day were selected and labeled. Samples were collected at 10, 20, 30, and 40 days after flowering, as well as at maturity, and the grains in each panicle were divided into superior and inferior grains. A portion of the grains was rapidly frozen in liquid nitrogen and then stored at −70 °C in an ultra-low temperature freezer for the determination of enzyme activities related to carbon and nitrogen metabolism.

Enzyme activities were determined according to the instructions provided with the assay kits (Shanghai Enzyme-linked Biotechnology Co., Ltd., Shanghai, China). The enzymes measured included carbon metabolism-related enzymes (AGPase, GBSS, SSS, SBE, and DBE) and nitrogen metabolism-related enzymes (GS and GOGAT).

#### 2.3.4. Starch and Protein Contents

Amylose content in brown rice samples was determined using the iodine-binding method described by Tan et al. [23]. Total starch content was measured using a Total Starch Assay Kit (Megazyme, Bray, Ireland) according to the manufacturer’s instructions. Amylopectin content was calculated as the difference between total starch content and amylose content.

The protein content of brown rice was determined by the Kjeldahl method using an automatic Kjeldahl nitrogen analyzer (FOSS, Kjeltec 8400). The nitrogen content was converted to protein content using a conversion factor of 5.95.

#### 2.3.5. Appearance Quality

A rice hulling machine (JLG-II, Taizhou Grain Instrument Factory, Taizhou, China) was used to remove rice husks. Bran powder was removed using a rice milling machine (JNMJ3, Taizhou Grain Instrument Factory, China). Rice chalkiness was determined using a chalky white scanner (Hangzhou Wanshen Test Technology Corporation, Hangzhou, China) according to GB/T 17891-2017 [24]. A total of 15 g of milled rice was spread evenly on the scanner surface without overlapping. The rice samples were scanned using Microtek-Scanwizard EZ2.2 software. The background was set to red before analysis, and the chalkiness parameters were obtained automatically.

The rice samples were placed in a 1 cm thick colorimetric dish. The dish was then illuminated in transmission mode using a colorimeter (Konica Minolta CM-5, Tokyo, Japan) with a D65 light source (simulated natural light). The Y-index of the colorimeter represents the transmittance and was used to indicate rice transparency.

### 2.4. Data Analysis

All data were subjected to analysis of variance (ANOVA) using SPSS Statistics software (version 19.0, IBM Corp., Armonk, NY, USA). Significant differences among treatments were evaluated using the least significant difference (LSD) test at *p* < 0.05. Graphical representations were generated using OriginPro (version 2021, OriginLab Corp., Northampton, MA, USA).

## 3. Results

### 3.1. Differences in Carbon Metabolism During the Grain-Filling Stage of Southern Soft and Non-Soft Japonica Rice

#### 3.1.1. Flag Leaf Net Photosynthetic Rate

**Figure 1:**
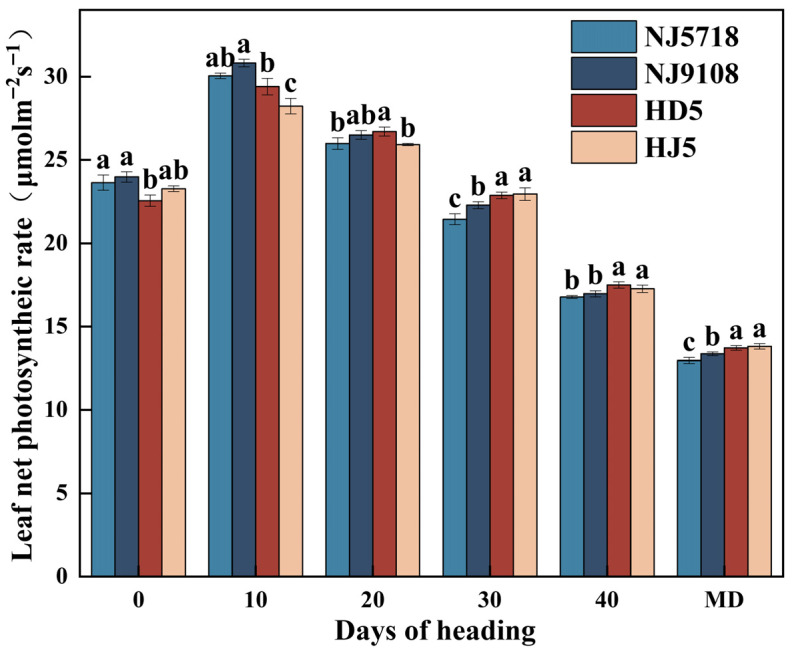
Difference in net photosynthetic rate off lag leaf between soft and non-soft japonica rice. Different letters within the same period indicate significant differences (*p* < 0.05).

The net photosynthetic rate of the flag leaf in both soft and non-soft japonica rice increased initially and then declined during grain filling, and the comparison between the two rice types varied with grain-filling stage (Figure 1). Compared with non-soft japonica rice, the net photosynthetic rate of the flag leaf in soft japonica rice was 1.53–6.30% higher at the heading stage and 2.18–9.18% higher at 10 days after flowering. No significant differences were observed at 20 days after flowering. From 30 days after flowering to maturity, the net photosynthetic rate was 2.59–6.59%, 1.73–4.15%, and 2.56–6.17% lower, respectively.

#### 3.1.2. SPAD of Upper Three Leaves

**Figure 2:**
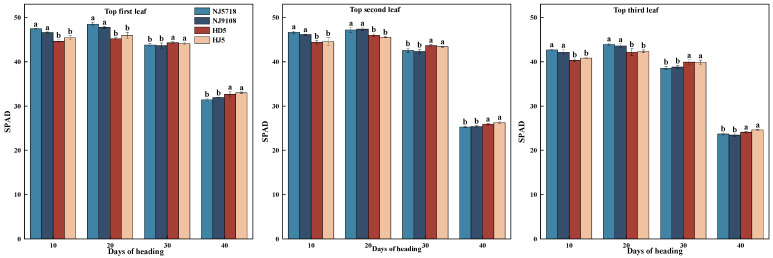
Differences in SPAD values of the top three leaves between soft and non-soft japonica rice. Different letters within the same period indicate significant differences (*p* < 0.05).

The SPAD values of the upper three leaves in both rice types increased initially and then declined after flowering, with soft japonica rice showing higher values at earlier stages but lower values at later stages (Figure 2).

Compared with non-soft japonica rice, the SPAD values of the top leaf in soft japonica rice were 2.50–6.24% and 3.88–7.25% higher from 10 to 20 days after flowering, and 0.72–1.56% and 2.22–4.79% lower from 30 to 40 days after flowering, respectively. The SPAD values of the second leaf were 3.50–5.16% and 2.65–4.03% higher from 10 to 20 days after flowering, and 1.99–2.91% and 1.97–3.70% lower from 30 to 40 days after flowering, respectively. The SPAD values of the third leaf were 3.15–5.83% and 2.96–4.26% higher from 10 to 20 days after flowering, and 2.68–3.44% and 1.79–4.65% lower from 30 to 40 days after flowering, respectively.

#### 3.1.3. Accumulation and Transport of NSC in Various Organs

NSC accumulation and transport in soft japonica rice were generally lower than in non-soft japonica rice, with differences across various organs observed at both the heading stage and maturity (Table 2). Compared with non-soft japonica rice, NSC accumulation in the stem, leaf, and panicle of soft japonica rice at the heading stage was 3.97–8.73%, 4.08–11.00%, and 3.00–12.13% lower, respectively. At maturity, NSC accumulation in the leaf and panicle of soft japonica rice was 2.00–7.22% and 1.56–22.69% lower, respectively. NSC transport in the stem and leaf, as well as NSC accumulation increase in the panicle of soft japonica rice, were 4.11–23.29%, 19.82–56.69%, and 1.35–23.98% lower, respectively.

#### 3.1.4. Dynamic Changes in NSC in Superior and Inferior Grains

**Figure 3:**
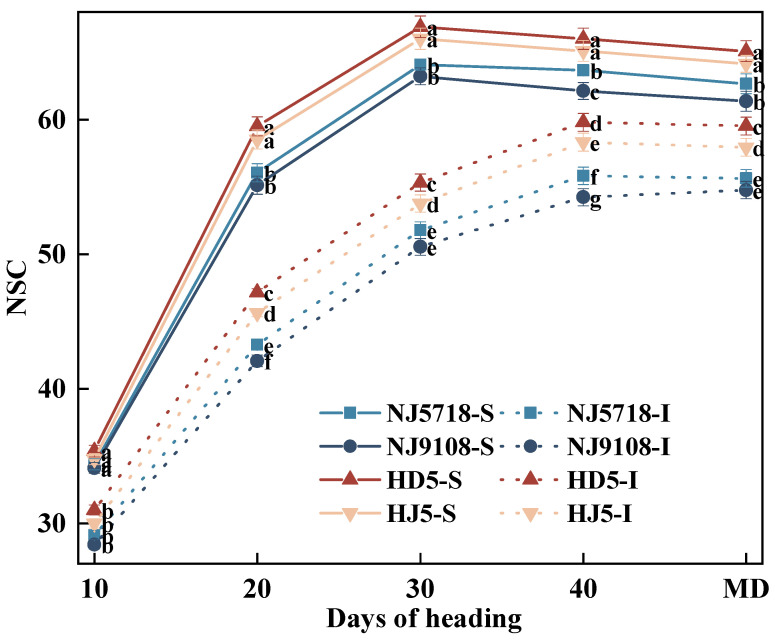
Differences in NSC dynamics of superior and inferior grains between soft and non-soft japonica rice. Values in the same column with different letters are significantly different (*p* < 0.05).

The NSC content in both superior and inferior grains was generally lower in soft japonica rice compared to non-soft japonica rice during grain filling, with inferior grains consistently showing lower levels than superior grains (Figure 3). Compared with the superior grains of non-soft japonica rice, NSC content in the superior grains of soft japonica rice was 1.13–3.55%, 4.22–7.35%, 2.89–5.48%, 2.24–5.87%, and 2.31–5.71% lower from 10 days after flowering to maturity. Similarly, compared with the inferior grains of non-soft japonica rice, NSC content in the inferior grains of soft japonica rice was 2.90–8.28%, 5.16–10.81%, 3.68–8.62%, 4.31–9.30%, and 3.96–8.02% lower during the same period.

Within soft japonica rice, NSC content in inferior grains was 14.63–17.33%, 21.50–24.94%, 18.10–21.13%, 10.18–14.79%, and 9.33–12.62% lower than in superior grains. Within non-soft japonica rice, NSC content in inferior grains was 10.88–15.20%, 19.40–23.31%, 16.19–19.64%, 8.15–11.64%, and 7.20–10.98% lower than in superior grains from 10 days after flowering to maturity.

#### 3.1.5. Activity of Carbon Metabolism-Related Enzymes in Superior and Inferior Grains

**Figure 4:**
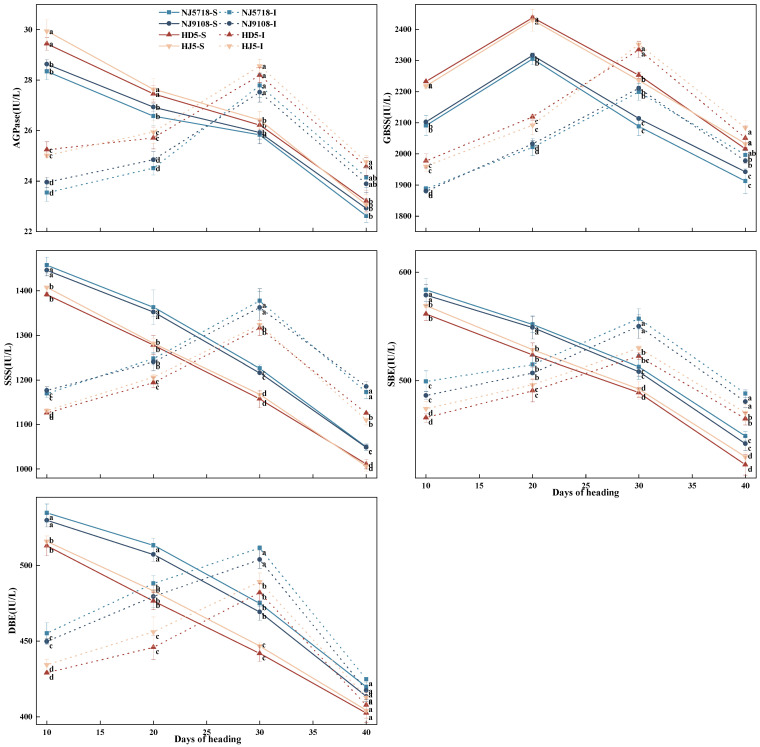
Differences in enzyme activities related to carbon metabolism and synthesis in superior and inferior grains between soft and non-soft japonica rice. Values in the same column with different letters are significantly different (*p* < 0.05).

Enzyme activities related to carbon metabolism exhibited temporal variation between superior and inferior grains, and soft japonica rice showed distinct differences compared with non-soft japonica rice, including lower AGPase and GBSS activities but higher SSS, SBE, and DBE activities. As shown in Figure 4, during the grain-filling period after flowering, the activities of AGPase, SSS, SBE, and DBE in the superior grains of the tested varieties showed a decreasing trend. GBSS activity first increased and then decreased. In contrast, enzyme activities in inferior grains showed a pattern of first increasing and then decreasing, reaching peak values at 30 days after flowering.

Compared with the superior grains of non-soft japonica rice, the AGPase activity in the superior grains of soft japonica rice was 0.61–5.34% lower from 10 to 40 days after flowering. The GBSS activity was 3.66–7.34% lower. The SSS activity was 2.73–6.66% higher. The SBE activity was 1.70–6.26% higher. The DBE activity was 2.26–7.74% higher from 10 to 40 days after flowering. Compared with the inferior grains of non-soft japonica rice, the AGPase activity in the inferior grains of soft japonica rice was 1.47–6.74% lower from 10 to 40 days after flowering. The GBSS activity was 2.65–6.53% lower. The SSS activity was 2.80–6.74% higher. The SBE activity was 2.56–7.21% higher. The DBE activity was 1.13–9.48% higher from 10 to 40 days after flowering.

Within soft japonica rice, compared with the superior grains, the AGPase activity in the inferior grains was 6.50–17.78% lower from 10 to 20 days after flowering, and 4.20–7.57% higher from 30 to 40 days after flowering. The GBSS activity was 9.76–10.58% lower from 10 to 20 days after flowering, and 1.84–5.89% higher from 30 to 40 days after flowering. The SSS activity was 7.70–19.75% lower from 10 to 20 days after flowering, and 11.08–13.27% higher from 30 to 40 days after flowering. The SBE activity was 6.23–16.72% lower from 10 to 20 days after flowering, and 7.14–10.47% higher from 30 to 40 days after flowering. The DBE activity was 3.76–15.88% lower from 10 to 20 days after flowering, and 0.54–9.00% higher from 30 to 40 days after flowering. Within non-soft japonica rice, compared with the superior grains, the AGPase activity in the inferior grains was 5.50–16.42% lower from 10 to 20 days after flowering, and 5.90–8.96% higher from 30 to 40 days after flowering. The GBSS activity was 10.86–14.17% lower from 10 to 20 days after flowering, and 0.90–5.18% higher from 30 to 40 days after flowering. The SSS activity was 5.61–19.99% lower from 10 to 20 days after flowering, and 9.87–14.40% higher from 30 to 40 days after flowering. The SBE activity was 5.25–18.18% lower from 10 to 20 days after flowering, and 6.20–11.40% higher from 30 to 40 days after flowering. The DBE activity was 4.27–16.83% lower from 10 to 20 days after flowering, and 0.87–10.75% higher from 30 to 40 days after flowering.

#### 3.1.6. Dynamic Accumulation of Brown Rice Starch and Components in Superior and Inferior Grains

**Figure 5:**
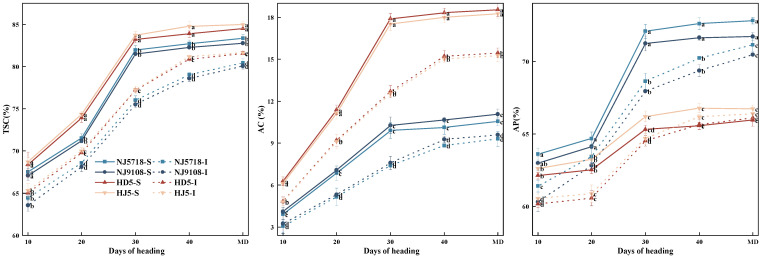
Dynamic accumulation of starch and its components in superior and inferior grains of soft and non-soft japonica rice. Values in the same column with different letters are significantly different (*p* < 0.05).

The dynamic changes in brown rice starch content in soft and non-soft japonica rice were generally consistent; however, significant differences were observed (Figure 5). Compared with the superior grains of non-soft japonica rice, the total starch content in the superior grains of soft japonica rice was 1.27–4.23% lower from 10 days after flowering to maturity. The amylose content was 32.68–44.72% lower, while the amylopectin content was 0.56–10.73% higher during the same period. Compared with the inferior grains of non-soft japonica rice, the total starch content in the inferior grains of soft japonica rice was 0.86–3.17% lower from 10 days after flowering to maturity. The amylose content was 2.21–43.95% lower, while the amylopectin content was 0.40–7.60% higher during the same period.

Within soft japonica rice, compared with the superior grains, the total starch content in the inferior grains was 1.82–5.89% lower from 10 days after flowering to maturity. The amylose content was 8.55–28.04% lower, while the amylopectin content was 0.81–5.73% lower during the same period. Within non-soft japonica rice, compared with the superior grains, the total starch content in the inferior grains was 3.21–5.41% lower from 10 days after flowering to maturity. The amylose content was 15.35–29.88% lower, while the amylopectin content was 0.93–3.93% lower from 10 to 30 days after flowering.

### 3.2. Differences in Nitrogen Metabolism During the Grain-Filling Stage of Southern Soft and Non-Soft Japonica Rice

#### 3.2.1. Accumulation and Transport of Nitrogen in Various Organs

The accumulation and transport of nitrogen in both soft and non-soft japonica rice showed differences across growth stages, with soft japonica rice generally exhibiting higher nitrogen levels at the heading stage and greater translocation in later stages compared with non-soft japonica rice (Table 3). Compared with non-soft japonica rice, the nitrogen accumulation in the leaves and panicles of soft japonica rice at the heading stage was 1.86–8.04% and 4.92–10.24% higher, respectively. At maturity, the nitrogen accumulation in the stems and panicles of soft japonica rice was 5.24–11.67% and 2.01–15.08% higher, respectively. In addition, nitrogen translocation from leaves was 5.37–11.87% higher, and nitrogen accumulation in the panicles was 7.36–13.38% higher in soft japonica rice.

#### 3.2.2. Dynamic Changes of Nitrogen in Superior and Inferior Grains

**Figure 6:**
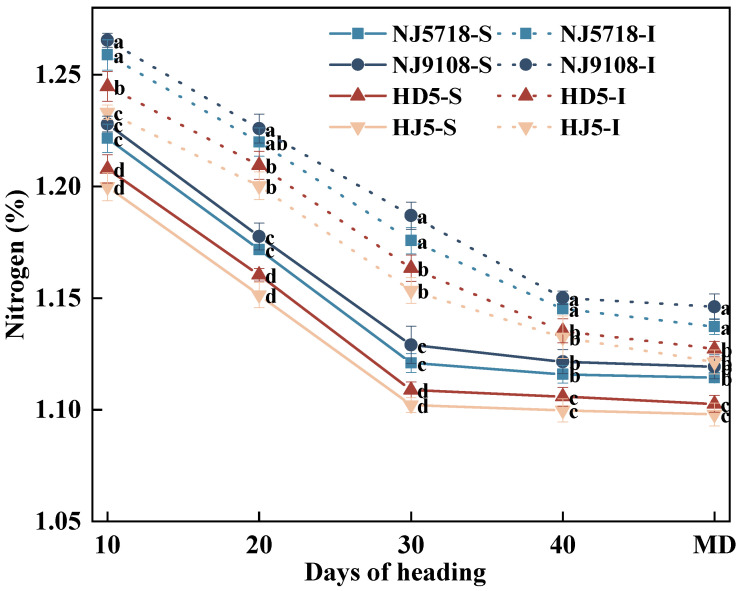
Differences in nitrogen dynamics between superior and inferior grains of soft and non-soft japonica rice. Values in the same column with different letters are significantly different (*p* < 0.05).

Compared with the superior grains of non-soft japonica rice, the nitrogen content in the superior grains of soft japonica rice was 0.91–2.34% higher from 10 days after flowering to maturity. Similarly, compared with the inferior grains of non-soft japonica rice, the nitrogen content in the inferior grains of soft japonica rice was 0.86–2.91% higher during the same period (Figure 6).

In soft japonica rice, the nitrogen content in the inferior grains was 1.61–5.89% higher than in the superior grains over the same period. Similarly, in non-soft japonica rice, the nitrogen content in the inferior grains was 1.70–5.57% higher than in the superior grains from 10 days after flowering to maturity.

#### 3.2.3. Activity of Enzymes Related to Nitrogen Metabolism Synthesis in Superior and Inferior Grains

**Figure 7:**
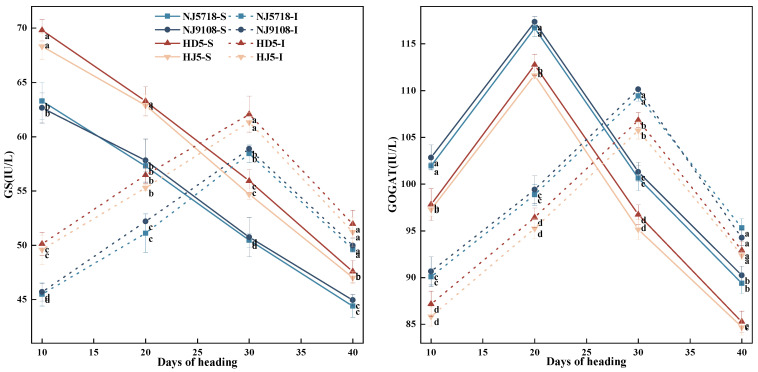
Differences in activities of nitrogen metabolism-related enzymes between superior and inferior grains of soft and non-soft japonica rice. Values in the same column with different letters are significantly different (*p* < 0.05).

As shown in Figure 7, during grain development after flowering, GS activity in superior grains of the tested varieties gradually decreased, whereas GOGAT activity first increased and then decreased. A similar trend was observed in inferior grains, with peak activity occurring at 30 days after flowering for both grain types.

Compared with the superior grains of non-soft japonica rice, the GS activity in the superior grains of soft japonica rice was 4.44–10.24% lower from 10 to 40 days after flowering, while the GOGAT activity was 3.48–6.53% higher during the same period. Compared with the inferior grains of non-soft japonica rice, the GS activity in the inferior grains of soft japonica rice was 2.44–9.48% lower from 10 to 40 days after flowering, while the GOGAT activity was 1.46–5.60% higher during the same period.

Within soft japonica rice, compared with the superior grains, the GS activity in the inferior grains was 8.91–28.12% lower from 10 to 20 days after flowering, and 10.47–16.67% higher from 30 to 40 days after flowering. The GOGAT activity was 11.06–15.75% lower from 10 to 20 days after flowering, and 4.47–9.43% higher from 30 to 40 days after flowering. Within non-soft japonica rice, compared with the superior grains, the GS activity in the inferior grains was 10.19–28.92% lower from 10 to 20 days after flowering, and 7.65–13.41% higher from 30 to 40 days after flowering. The GOGAT activity was 10.40–15.52% lower from 10 to 20 days after flowering, and 8.35–12.28% higher from 30 to 40 days after flowering.

#### 3.2.4. Dynamic Accumulation of Brown Rice Protein in Superior and Inferior Grains

**Figure 8:**
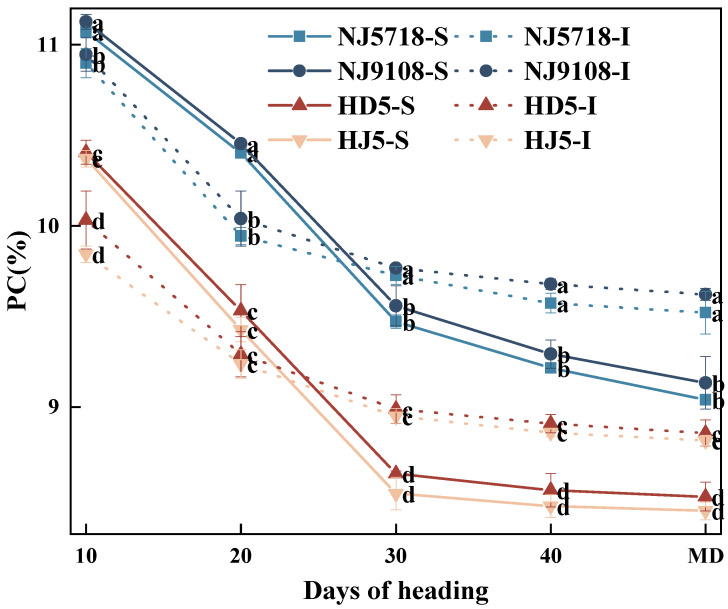
Differences in the dynamics of protein content accumulation between superior and inferior grains of soft and non-soft japonica rice. Values in the same column with different letters are significantly different (*p* < 0.05).

Protein accumulation patterns in brown rice were generally consistent between soft and non-soft japonica rice, though significant differences were observed between superior and inferior grains (Figure 8). Compared with the superior grains of non-soft japonica rice, the protein content in the superior grains of soft japonica rice was 6.28–12.15% higher from 10 days after flowering to maturity. Compared with the inferior grains of non-soft japonica rice, the protein content in the inferior grains of soft japonica rice was 7.03–11.19% higher during the same period.

In soft japonica rice, the protein content in inferior grains was 1.14–4.87% lower from 10 to 20 days after flowering, but 1.75–6.44% higher from 30 days after flowering to maturity. In non-soft japonica rice, the protein content in inferior grains was 1.46–5.40% lower from 10 to 20 days after flowering, but 3.65–5.46% higher from 30 days after flowering to maturity.

### 3.3. Differences in Appearance Quality of Superior and Inferior Grains of Southern Soft and Non-Soft Japonica Rice

**Figure 9:**
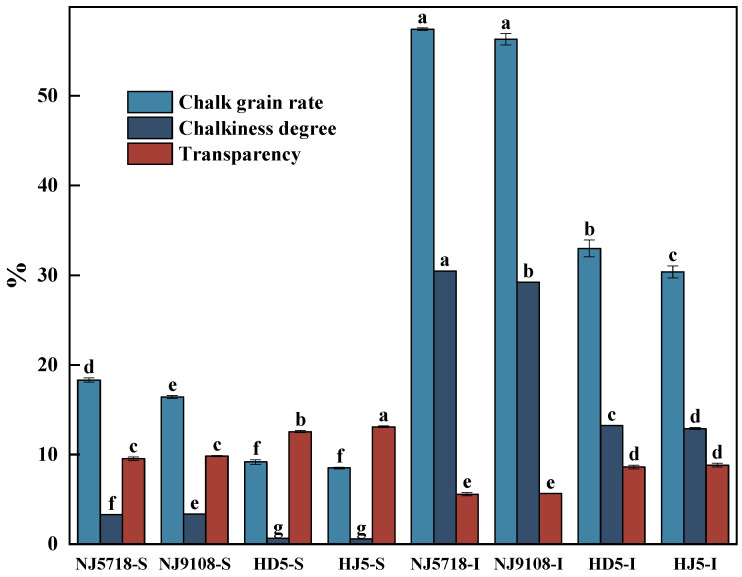
Differences in chalkiness and transparency between superior and inferior grains of soft and non-soft japonica rice. Different letters within the same trait indicate significant differences at *p* < 0.05.

Significant differences in appearance quality were observed between superior and inferior grains of soft and non-soft japonica rice (Figure 9). Compared with the superior grains of non-soft japonica rice, the chalky grain rate and chalkiness degree of superior grains in soft japonica rice were 79.00–115.22% and 410.94–481.03% higher, respectively, with transparency being 21.67–27.27% lower. Compared with the inferior grains of non-soft japonica rice, the chalky grain rate and chalkiness degree of inferior grains in soft japonica rice were 70.72–89.17% and 121.03–136.31% higher, respectively, with transparency being 34.19–36.70% lower.

Within soft japonica rice, compared with the superior grains, the chalky grain rate and chalkiness degree of inferior grains were 67.51–71.43% and 88.47–89.26% higher, respectively, with transparency being 40.55–43.34% lower. Within non-soft japonica rice, compared with the superior grains, the chalky grain rate and chalkiness degree of inferior grains were 69.80–74.23% and 95.03–95.61% higher, respectively, with transparency being 29.88–34.30% lower.

### 3.4. Analysis of Relationship

#### 3.4.1. Correlation Analysis of Non-Structural Carbohydrates, Nitrogen, and Starch and Protein Contents

**Figure 10:**
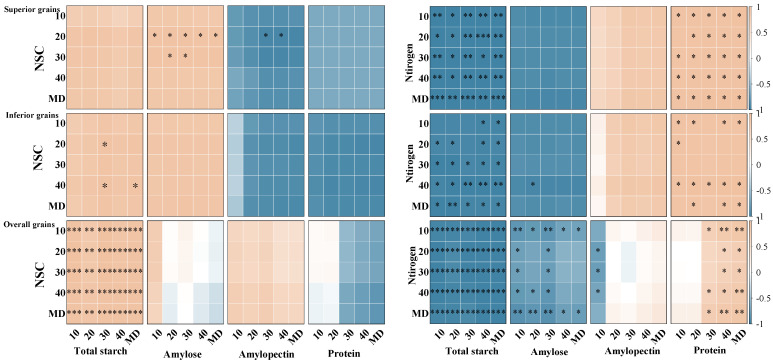
Correlation analysis of non-structural carbohydrates and nitrogen with starch and protein contents in grains of soft and non-soft japonica rice. Yellow represents a positive correlation and blue represents a negative correlation. The intensity of colors and * indicates correlation at the 0.05% level, and ** indicates correlation at the 0.01% level, and *** indicates correlation at the 0.001% level.

The correlation analysis showed that significant relationships existed between non-structural carbohydrate (NSC) and nitrogen contents and the accumulation of starch and protein in grains during the grain-filling stage, with clear differences between superior and inferior grains (Figure 10).

In superior grains, NSC content was significantly positively correlated with starch content from 10 to 30 days after flowering (DAF), whereas it showed negative or weak correlations with protein content. In the late grain-filling stage (40 DAF), the correlation between NSC and starch content weakened. Nitrogen content was significantly positively correlated with protein content throughout the grain-filling period, while it showed negative or weak negative correlations with starch content.

In inferior grains, NSC content also showed a positive correlation with starch content, although the correlation was generally weaker than that in superior grains and exhibited greater fluctuation in the early grain-filling stage. Meanwhile, NSC content was mostly negatively correlated with protein content. Nitrogen content remained significantly positively correlated with protein content at all grain-filling stages, while it showed negative or weak negative correlations with starch content.

At the overall grain level, NSC content was generally positively correlated with starch content and negatively correlated with protein content. Nitrogen content showed a significant positive correlation with protein content but was mostly negatively correlated with starch content.

#### 3.4.2. Correlation Analysis of Carbon and Nitrogen Metabolism-Related Enzymes with Starch and Protein Contents

**Figure 11:**
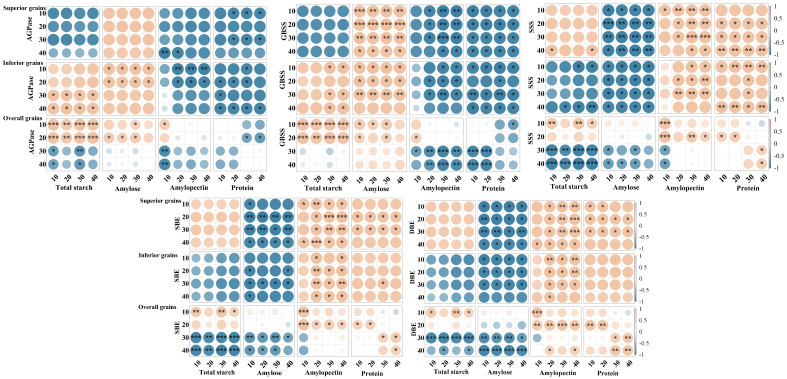

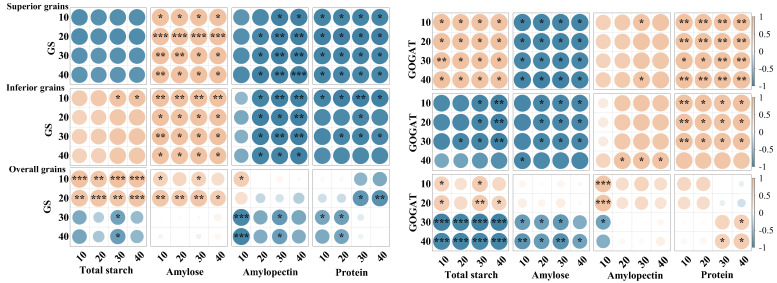
Correlation analysis of carbon and nitrogen metabolism-related enzymes with starch and protein contents in grains of soft and non-soft japonica rice. Yellow represents a positive correlation and blue represents a negative correlation. The in-tensity of colors and * indicates correlation at the 0.05% level, and ** indicates correlation at the 0.01% level, and *** indicates correlation at the 0.001% level.

Correlation analysis indicated that significant relationships existed between the activities of carbon and nitrogen metabolism-related enzymes and the contents of starch and protein in grains during the grain-filling stage, although these relationships varied with grain position and grain-filling stage (Figure 11).

In superior grains, the activities of AGPase and GBSS were generally negatively correlated with total starch content. In contrast, the activities of SSS, SBE, and DBE showed certain correlations with starch and its components, although the direction and strength of these correlations varied among different grain-filling stages. Carbon metabolism-related enzyme activities were mostly negatively or weakly correlated with protein content. In comparison, the activities of the nitrogen metabolism-related enzymes GS and GOGAT were significantly positively correlated with protein content but were mostly negatively or weakly negatively correlated with starch content.

In inferior grains, the activities of AGPase and GBSS were generally positively correlated with total starch content. The activities of SSS, SBE, and DBE also showed correlations with starch and its components, although the correlation strength varied across different grain-filling stages. The activities of GS and GOGAT remained significantly positively correlated with protein content but were mostly negatively or weakly negatively correlated with starch content.

At the overall grain level, the relationships between carbon metabolism-related enzyme activities and starch content exhibited clear stage-dependent characteristics. These enzyme activities were generally positively correlated with starch content during the early and middle grain-filling stages, whereas the correlations weakened or even turned negative during the late grain-filling stage. Meanwhile, nitrogen metabolism-related enzyme activities were generally positively correlated with protein content but mostly negatively correlated with starch content.

#### 3.4.3. Correlation Analysis of Chalkiness, Transparency, and Starch and Protein Contents

**Figure 12:**
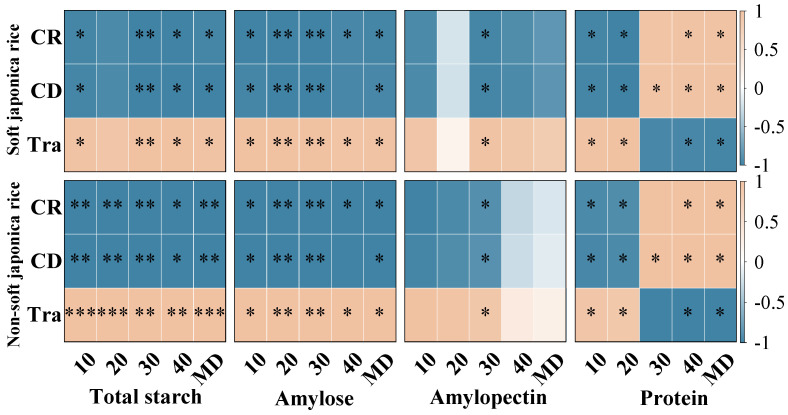
Correlation analysis between appearance quality and starch and protein contents in superior and inferior grains of soft and non-soft japonica rice. Yellow represents a positive correlation and blue represents a negative correlation. The intensity of colors and * indicates correlation at the 0.05% level, and ** indicates correlation at the 0.01% level, and *** indicates correlation at the 0.001% level.

Correlation analysis revealed significant relationships between grain appearance quality and the contents of starch and protein, with certain differences between soft and non-soft japonica rice (Figure 12).

Overall, the chalky grain rate (CD) and chalkiness degree (CR) were generally negatively correlated with total starch and amylose contents but positively correlated with protein content. In contrast, transparency (Tra) showed positive correlations with total starch and amylose contents but negative correlations with protein content.

In soft japonica rice, the negative correlations between chalkiness traits and starch components were more pronounced, and their positive correlations with protein content were stronger. Meanwhile, transparency showed a relatively strong positive correlation with starch content. In comparison, the correlations between appearance quality traits and starch and protein contents in non-soft japonica rice were relatively weaker, although the overall trends were similar.

## 4. Discussion

### 4.1. Differences in Carbon and Nitrogen Metabolism Between Superior and Inferior Grains of Southern Soft and Non-Soft Japonica Rice

This study showed that southern soft japonica rice exhibited a distinct metabolic pattern during grain filling, characterized by relatively weaker carbon metabolism but stronger nitrogen metabolism than non-soft rice. The extent of this pattern differed between superior and inferior grains and was closely associated with starch biosynthesis, protein accumulation, and endosperm structural characteristics related to appearance quality formation [25,26].

In terms of carbon metabolism, soft japonica rice exhibited lower efficiency of carbon assimilate translocation. Although the photosynthetic rate of the flag leaf during the early grain-filling stage was slightly higher than that in non-soft rice (Figure 1), this advantage did not result in greater final carbon accumulation in the grains (Table 1; Figure 1, Figure 2 and Figure 3). Specifically, the accumulation of non-structural carbohydrates (NSC) in the stems, leaves, and panicles of soft japonica rice was lower than that in non-soft rice, particularly at the heading and maturity stages (Table 2; Figure 3). This insufficiency in carbon supply became more pronounced during grain filling, especially in inferior grains, indicating a relatively weaker capacity for assimilate remobilization in soft japonica rice [27,28]. Because NSC stored in vegetative organs serves as an important transient carbon reserve for grain filling, reduced NSC accumulation and remobilization may impair assimilate supply to developing grains [29]. Previous studies have shown that inadequate carbohydrate supply during grain filling restricts endosperm filling and promotes chalkiness formation, particularly in inferior grains. In line with this, the lower NSC levels observed in soft japonica rice may have contributed to impaired starch deposition and greater chalkiness in inferior grains [7,30]. The activities of key starch-synthesis enzymes also differed between soft and non-soft rice grains. Specifically, the activities of AGPase and GBSS were lower, whereas those of SSS, SBE, and DBE were higher in soft rice grains (Figure 4). These changes suggest that amylose synthesis was restricted during starch biosynthesis in soft japonica rice, whereas processes related to amylopectin synthesis and modification were relatively more active [11]. This characteristic was particularly pronounced in inferior grains [11,31]. Together, these results suggest that soft japonica rice differs from non-soft rice not only in carbon supply but also in the intrinsic regulation of carbon metabolism [32,33].

In terms of nitrogen metabolism, soft japonica rice exhibited stronger and more sustained nitrogen assimilation capacity. During the early grain-filling stage, the higher SPAD value of leaves in soft japonica rice (Figure 2) suggested a greater capacity for nitrogen assimilation [34]. At the heading stage, vegetative organs, especially leaves, accumulated more nitrogen (Table 3), which was subsequently remobilized to the grains during grain filling. Meanwhile, GOGAT activity remained higher than that in non-soft rice throughout grain filling (Figure 7), thereby sustaining the GS-GOGAT cycle, amino acid production [35], and protein synthesis (Figure 8). Even in inferior grains under limited carbon supply, nitrogen content and GOGAT activity remained relatively high (Table 3; Figure 7), suggesting that nitrogen metabolism in soft japonica rice may be relatively less sensitive to carbon limitation [36]. This may be associated with sustained GS-GOGAT activity together with continued nitrogen supply from vegetative remobilization and possibly post-anthesis uptake, which may help maintain nitrogen metabolism under carbon-limited conditions [7].

Therefore, carbon and nitrogen metabolism in soft japonica rice do not function independently but interact in a complex manner. This study further suggested that increased nitrogen metabolic activity in inferior grains may be accompanied by a relative decline in the activities of SBE and DBE (Figure 4). This phenomenon suggests that potential competition for substrates and energy may exist between carbon and nitrogen metabolism [37]. When limited resources and energy are preferentially allocated to nitrogen assimilation and protein synthesis, the substrate and energy supply available for maintaining carbon metabolism-related enzyme activity is relatively reduced, thereby imposing constraints on starch synthesis [32,38]. This pattern may reflect differential regulatory sensitivity of carbon and nitrogen metabolism in inferior grains under carbon-limited conditions, with nitrogen assimilation and storage protein synthesis retaining a relatively higher developmental priority, whereas starch biosynthesis appears more susceptible to reduced assimilate supply [39,40]. This competitive relationship may be particularly pronounced in inferior grains, where carbon supply is already limited, and may disrupt the continuity and stability of grain filling, thereby affecting grain-filling performance and final grain quality [41].

### 4.2. Effects of Carbon and Nitrogen Metabolism Differences on the Formation of Appearance Quality in Superior and Inferior Grains of Rice

It should also be noted that the four cultivars differed in agronomic traits such as growth-period type, spikelet number per panicle, and 1000-grain weight (Table 1). These cultivar-dependent agronomic differences may affect grain-filling patterns and assimilate allocation within the panicle, thereby contributing to the positional variation in chalky appearance observed in this study [42]. The characteristics of carbon and nitrogen metabolism in soft japonica rice were closely associated with the formation of appearance quality, particularly grain chalkiness and transparency. Differences in carbon metabolism during grain filling, especially those related to starch synthesis, were associated with reduced amylose synthesis and relatively enhanced amylopectin synthesis in soft japonica rice grains (Figure 5). This shift may affect the arrangement of starch granules, resulting in increased intergranular spaces and a more irregular packing pattern, which may ultimately promote chalkiness formation [43]. Moreover, significant differences were observed between superior and inferior grains in starch accumulation dynamics during grain filling. Correlation analysis showed that the chalky grain rate was significantly positively correlated with starch accumulation during the early grain-filling stage, particularly in inferior grains, suggesting that abnormal early starch deposition patterns may be associated with chalkiness formation (Figure 10, Figure 11 and Figure 12). This relationship may also be associated with differences in NSC availability during grain filling, because insufficient reserve carbohydrate supply can limit starch deposition in developing grains, particularly in inferior grains, thereby promoting chalkiness formation [30].

Carbon metabolism may provide the fundamental structural basis of starch during endosperm development, whereas nitrogen metabolism may further influence the structural organization of the endosperm [44]. During the late grain-filling stage, especially in inferior grains, although carbon supply became limited, relatively high levels of protein still accumulated (Figure 8). Furthermore, these proteins were unevenly distributed within the endosperm, leading to structural heterogeneity [3]. Localized excessive protein deposition may disrupt the regular arrangement of starch granules, increase light scattering within the endosperm, and consequently reduce grain transparency while increasing chalkiness [45]. Correlation analysis also showed that chalkiness was significantly positively correlated with protein content during the middle and late grain-filling stages, particularly in inferior grains, further supporting the important role of nitrogen metabolism in the formation of appearance quality (Figure 12). Environmental factors during grain filling, especially light conditions and stress, may also strongly influence grain chalkiness by modifying carbon and nitrogen metabolism [29]. In particular, weak light has been reported to reduce photosynthetic carbon assimilation, sucrose transport, and starch synthesis while disturbing nitrogen assimilation and translocation, thereby affecting starch and protein deposition and final grain appearance [46,47].

In summary, the appearance quality of soft japonica rice was closely associated with differences in carbon and nitrogen metabolism between superior and inferior grains. Carbon metabolism may contribute to the structural basis of endosperm tissue by affecting starch synthesis rate and compositional characteristics, whereas nitrogen metabolism may further influence endosperm structure through protein synthesis and accumulation. The imbalance between carbon and nitrogen metabolism in inferior grains may be an important factor contributing to the enhanced positional differences in appearance quality within the panicle. However, the present study mainly provides correlative physiological evidence based on the relationships among NSC and nitrogen accumulation, metabolic enzyme activities, starch and protein deposition, and appearance quality traits, and therefore does not establish direct cause–effect relationships. Thus, the proposed mechanisms should be interpreted with caution, and further molecular, genetic, and targeted physiological studies are needed to verify the causal links involved. Nevertheless, these findings provide useful physiological evidence for understanding grain quality formation in soft rice and may offer practical implications for future breeding improvement and cultivation management, as agronomic strategies such as optimized nitrogen fertilization, appropriate irrigation management during grain filling, and cultivation practices that improve canopy light interception and promote more uniform grain filling may help mitigate chalkiness formation by stabilizing source–sink balance, enhancing assimilate translocation, and reducing positional differences in grain quality within the panicle [48].

## 5. Conclusions

This study revealed significant differences in carbon and nitrogen metabolism during the grain-filling stage between southern soft and non-soft japonica rice, and clarified the relationships between these metabolic differences and the formation of rice appearance quality. Compared with non-soft japonica rice, soft japonica rice showed relatively weaker carbon metabolism during grain filling, as indicated by lower accumulation and translocation of NSC, which was more pronounced in inferior grains. In contrast, nitrogen metabolism in soft japonica rice was relatively stronger, characterized by higher activities of nitrogen assimilation enzymes and higher protein content. Carbon and nitrogen metabolism exhibited different patterns of coordination and competition between superior and inferior grains, thereby altering the accumulation characteristics of starch and protein in the grains and further affecting the formation of appearance quality. Compared with non-soft japonica rice, soft japonica rice showed significantly higher chalky grain rate and chalkiness degree, but lower transparency. Overall, the coordination between carbon and nitrogen metabolism during the grain-filling stage constitutes an important physiological basis affecting the formation of appearance quality in soft japonica rice. These findings provide a theoretical basis for further understanding the mechanisms underlying appearance quality formation in soft japonica rice and for developing genetic improvement and cultivation strategies.

## Figures and Tables

**Table 1:** Differences in agronomic traits between soft and non-soft japonica rice.

Cultivar	Growth-Period Type	Spikelets Per Panicle	1000-Grain Weight (g)
NJ5718	Medium-maturing medium-grain japonica rice	124.71 ± 4.62 c	30.82 ± 0.18 a
NJ9108	Late-maturing medium-grain japonica rice	165.55 ± 8.27 a	23.29 ± 0.24 d
HD5	Late-maturing medium-grain japonica rice	128.57 ± 7.84 bc	27.38 ± 0.10 b
HJ5	Medium-maturing medium-grain japonica rice	134.75 ± 5.94 b	27.05 ± 0.09 c

Values in the same column with different letters are significantly different (*p* < 0.05).

**Table 2:** Differences in NSC accumulation and transport between soft and non-soft japonica rice at different stages (kg hm^−2^).

Cultivar	NSC Accumulation						Stem NSC Transport	Leaf NSC Transport	Panicle NSC Increment
	Heading			Maturity					
	Stem	Leaf	Panicle	Stem	Leaf	Panicle			
NJ5718	1826.70 ± 11.32 b	377.55 ± 4.05 c	344.40 ± 4.63 b	1004.40 ± 2.30 a	361.05 ± 5.34 b	2765.10 ± 12.48 d	822.30 ± 4.15 d	16.50 ± 2.01 c	2420.70 ± 7.68 d
NJ9108	1883.55 ± 13.52 b	384.45 ± 4.77 c	368.70 ± 5.01 b	899.70 ± 1.95 b	358.35 ± 0.74 c	3018.15 ± 4.65 c	983.85 ± 2.23 c	26.10 ± 0.65 b	2649.45 ± 10.99 c
HD5	1961.40 ± 19.02 a	400.80 ± 3.44 b	391.95 ± 3.29 a	889.50 ± 1.56 b	368.40 ± 7.62 b	3576.45 ± 4.58 a	1071.90 ± 5.55 a	32.55 ± 1.45 a	3184.50 ± 4.17 a
HJ5	2001.45 ± 20.35 a	424.20 ± 5.23 a	380.10 ± 2.21 a	975.45 ± 1.87 a	386.25 ± 6.48 a	3065.85 ± 10.30 b	1026.00 ± 3.79 b	38.10 ± 1.67 a	2685.75 ± 13.51 b

Values in the same column with different letters are significantly different (*p* < 0.05).

**Table 3:** Differences in nitrogen accumulation and transport between soft rice and non-soft rice japonica rice at different stages (kg hm^−2^).

Cultivar	Nitrogen Accumulation						Stem N Transport	Leaf N Transport	Panicle N Increment
	Heading			Maturity					
	Stem	Leaf	Panicle	Stem	Leaf	Panicle			
NJ5718	62.96 ± 0.56 a	82.05 ± 0.46 b	18.3 ± 0.42 b	38.15573364 ± 0.32 a	25.5 ± 0.25 b	127.65 ± 0.28 b	44.655 ± 0.46 a	56.55 ± 0.24 a	109.35 ± 0.45 b
NJ9108	67.95 ± 0.34 c	80.55 ± 0.45 a	19.05 ± 0.17 a	41.727759768 ± 0.32 c	24.6 ± 0.24 ab	130.5 ± 0.43 a	48.9 ± 0.3 c	55.95 ± 0.31 a	111.45 ± 0.48 a
HD5	61.14 ± 0.65 c	75.45 ± 0.22 c	17.1 ± 0.16 d	36.856815048 ± 0.11 c	24.9 ± 0.23 ab	115.4 ± 0.54 c	44.04 ± 0.24 c	50.55 ± 0.23 b	98.3 ± 0.65 d
HJ5	64.20 ± 0.24 b	79.05 ± 0.45 c	17.40 ± 0.14 c	40.266476352 ± 0.35 b	25.95 ± 0.36 a	119.25 ± 0.3 b	46.8 ± 0.32 b	53.1 ± 0.29 b	101.85 ± 0.51 c

Values in the same column with different letters are significantly different (*p* < 0.05).

## Data Availability

The original contributions presented in this study are included in the article. Further inquiries can be directed to the corresponding author.
